# An Alternate Technique for Goniotomy: Description of Procedure and Preliminary Results

**DOI:** 10.18502/jovr.v17i2.10787

**Published:** 2022-04-29

**Authors:** Thomas Shute, Wesley Green, James Liu, Arsham Sheybani

**Affiliations:** ^1^Department of Ophthalmology and Visual Sciences, Washington University School of Medicine, St. Louis, MO, USA; ^2^EYE-Q Vision Care, Fresno CA, USA; ^3^Davis Deuhr Dean Eye Care, Madison, WI, USA

**Keywords:** Glaucoma, Goniotomy, Microinvasive Glaucoma Surgery

## Abstract

**Purpose:**

Multiple glaucoma treatment modalities seek to lower IOP by bypassing or removing a portion of the juxtacanalicular trabecular meshwork. These procedures often require expensive implants or specialized surgical instruments. The authors developed a technique for ab interno goniectomy utilizing a standard disposable 25-gauge hypodermic needle. The surgical procedure—termed bent ab interno needle goniectomy (BANG)—and preliminary results are presented here.

**Methods:**

A retrospective chart review was performed for all patients who underwent goniotomy using a modified hypodermic needle by one of the three authors between July 2017 and June 2018. The mean and standard deviation pre- and postoperative IOP and the number of glaucoma medications were calculated. The student paired *t-*test was used to compare pre- and postoperative data. A *P*-value of < 0.05 was considered statistically significant.

**Results:**

At postoperative month six, the mean IOP was 13.3 ± 2.5 mmHg (*P* = 3.6 × 10 ${}^{-7}$ ) on 0.5 ± 0.8 topical glaucoma medications (*P* = 0.01). A ≥ 20% reduction in IOP was achieved in 73% of patients. Seventy-three percent of patients required ≥ 1 fewer medication, while 73% of patients required no medications for IOP control. Forty-one percent of those treated achieved IOP ≤ 12 mmHg.

**Conclusion:**

The BANG procedure is a low-cost MIGS technique available to surgeons around the world with preliminary outcomes similar to more expensive alternatives.

##  INTRODUCTION

Glaucoma is the leading cause of irreversible blindness worldwide, affecting more than 64 million people. By 2040, the number of individuals with glaucoma is expected to eclipse 110 million.^[1]^ Intraocular pressure (IOP) remains the only modifiable risk factor for disease progression. Decreasing IOP with medications, LASER, and incisional surgery are the only treatments proven to mitigate the risk of glaucomatous vision loss. These interventions aim to lower IOP by decreasing aqueous humor (AH) production or facilitating its outflow.

The major site of resistance to AH outflow is thought to be the juxtacanalicular trabecular meshwork (TM).^[2,3,4]^ Multiple treatment modalities seek to lower IOP by bypassing this tissue or attempting to remove it altogether.^[5,6,7,8,9,10,11,12,13]^ While several of these procedures have met with success in the short term, long-term outcomes in adults have been variable.^[14,15,16,17]^ There are many reasons a TM-based procedure may fail to achieve significant IOP lowering over time. In post-goniotomy patients, obstruction of aqueous outflow channels from scarring of residual tissue leaflets might play a role. Devices such as the Trabectome (NeoMedix, Tustin, CA USA) and Kahook Dual Blade (New World Medical, Rancho Cucamonga, CA USA) as well as techniques such as goniocurettage or excimer laser trabeculostomy seek to ablate or remove a portion of the TM entirely.^[8,10,16,18,19,20]^ While these techniques have shown promise, they often require expensive equipment or specialized instruments. Alternative, low-cost methods for goniotomy have been described, but these tend to incise or lacerate rather than completely excise a segment of TM.^[21,22]^ Without excision, leaflet scarring could lead to loss of effect over time. The authors developed a procedure for ab interno TM excision (goniectomy) utilizing a disposable 25-gauge hypodermic needle—termed bent ab interno needle goniectomy (BANG)—for the treatment of glaucoma. The surgical technique and preliminary results are presented here.

##  METHODS

The study complied with the Declaration of Helsinki and was approved by the Washington University in St. Louis institutional review board. A retrospective chart review was performed for all patients who underwent BANG by one of the three authors between July 2017 and December 2018. All patients provided written consent before surgery. Preoperative assessment included gonioscopy performed by one of the three authors to confirm identifiable landmarks—specifically TM—and absence of significant nasal peripheral anterior synechiae (PAS). An angle was deemed open if ≥ 180 degrees was ≥ Shaffer 1. The procedure was performed alone or in combination with cataract surgery in adults with open angle glaucoma. After excision, TM samples were preserved in 4% paraformaldehyde/phosphate-buffered saline before being processed for histology and embedded in paraffin. Sections were cut and stained with hematoxylin–eosin and periodic acid-Schiff.

The mean and standard deviation pre- and postoperative IOP were calculated and the number of glaucoma medications was tabulated for each patient. The student paired *t-*test was used to compare pre- and postoperative data. A *P*-value of < 0.05 was considered statistically significant.

**Figure 1:**
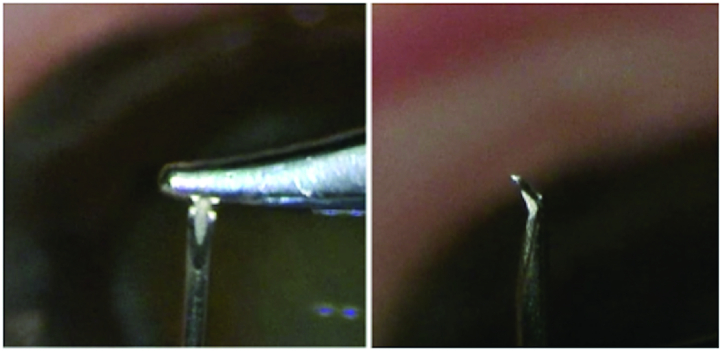
Fashioning a goniotome from a 25-gauge needle using a needle driver.

**Figure 2:**
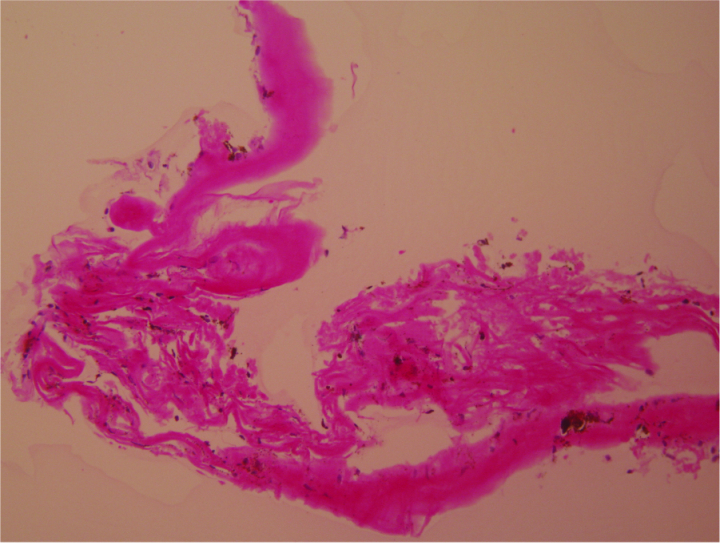
Histologic specimen of human trabecular meshwork following excision with a modified 25-gauge hypodermic needle. Light micrograph, hematoxylin–eosin, 200 × magnification.

**Table 1:** Patient demographics


** Ethnicity **			** OAG Severity**			** Sex**		** Avg age (yr)**
African–American	Latino	Caucasian	Mild	Mod	Sev	M	F	
4	6	13	23 eyes	7 eyes	11 eyes	11	12	76.6 ± 7.7
	
	

**Table 2:** Preoperative patient characteristics


** MD (dB) **	** PSD**	** RNFL thickness (um) **	** Shaffer Grade**	** Tmax (mmHg)**	** CCT (um)**	** Extent of treatment (degrees)**
–5.9 ± 7.8	4.3 ± 3.2	74.5 ± 15.1	1.75 ± 1	23.9 ± 6.1	549.6 ± 39.3	102.4 ± 8.1
	
	

**Table 3:** Comparison of IOP and medications pre- and post-BANG


** Pre-op **		** POM 1 **		** POM 3 **		** POM 6 **	
Mean IOP (mmHg)	Meds	Mean IOP (mmHg)	Meds	Mean IOP (mmHg)	Meds	Mean IOP (mmHg)	Meds
17.4 ± 4.1	1.1 ± 1.4	12.9 ± 2.6	0.4 ± 0.9	13.4 ± 2.5	0.4 ± 0.7	13.3 ± 2.5	0.5 ± 0.8
	*P*-value	1 × 10 ${}^{-6}$	0.01	3.5 × 10^5^	0.01	3.6 × 10 ${}^{-7}$	0.01
	
	

**Table 4:** Stratification of postoperative month six data


** Six Months Post-BANG **	
≥ 20% IOP reduction	30/41 eyes (73%)
IOP ≤ 12 mmHg	17/41 eyes (41%)
≥ 1 fewer medication	16/22 eyes (73%)
Zero medications	30/41 eyes (73%)
	
	

### Surgical Procedure

The operative eye was prepped and draped in the usual sterile ophthalmic fashion. A wire eyelid speculum was placed. A 1-mm paracentesis was created either inferiorly or superiorly, and viscoelastic was used to fill the anterior chamber. If performed in concert with cataract surgery, the temporal incision was used to gain access to the nasal angle. If performed as a standalone procedure, a 1.4 mm paracentesis was created temporally. The patient's head and operating microscope were rotated to aid visualization of the angle structures. A gonioprism was used to view the angle. A goniotome was fashioned by bending the distal 1 mm of a sterile 25-gauge 5/8 inch hypodermic needle toward the bevel using a needle driver [Figure 1]. The bent needle was used to excise the nasal 100 degrees of TM. The excised portion of TM was sent for histologic analysis. The viscoelastic was removed using either an irrigation–aspiration system or evacuated through a paracentesis using balanced salt solution (BSS) on a cannula. Each wound was verified watertight, and the surgeon's preference of antibiotic plus steroid was administered.

If combined with cataract surgery, BANG was performed before standard phacoemulsification. Postoperative care and follow-up proceeded as with standard phacoemulsification with intraocular lens implantation.

##  RESULTS

Forty-one eyes of 23 patients underwent the BANG procedure either alone (two eyes) or combined with phacoemulsification (39 eyes). All patients were classified as open-angle glaucoma via gonioscopy with stages ranging from mild to severe. Twelve of the twenty-three patients were female [Table 1]. The average preoperative mean deviation (MD) on Humphrey visual field testing was –5.9 ± 7.8 decibels (dB) and pattern standard deviation (PSD) was 4.3 ± 3.2. The average preoperative retinal nerve fiber layer (RNFL) thickness measured by optical coherence tomography (OCT) was 74.5 ± 15.1 microns. The mean Shaffer grade on gonioscopy was 1.75 ± 1. The average maximum IOP (Tmax) was 23.9 ± 6.1 mmHg, while the mean central corneal thickness (CCT) was 549.6 ± 39.3 microns. The mean treatment spanned 102.4 ± 8.1 degrees of the nasal TM [Table 2].

The average preoperative IOP was 17.4 ± 4.1 mmHg on 1.1 ± 1.4 topical glaucoma medications. Sixteen patients (22 eyes) were using at least one topical medication for IOP control preoperatively. At postoperative month (POM) one, the mean IOP was 12.9 ± 2.6 mmHg (*P* = 1 × 10 ${}^{-6}$ ) on 0.4 ± 0.9 topical glaucoma medications (*P* = 0.01). At POM three, the mean IOP was 13.4 ± 2.5 mmHg (*P* = 3.5 × 10 ${}^{-5}$ ) on 0.4 ± 0.7 topical glaucoma medications (*P* = 0.01). At POM six, the mean IOP was 13.3 ± 2.5 mmHg (*P* = 3.6 × 10 ${}^{-7}$ ) on 0.5 ± 0.8 topical glaucoma medications (*P* = 0.01) [Table 3]. A ≥ 20% reduction in IOP was achieved in 73% of patients. Seventy-three percent of them saw their drop burden decrease by ≥ 1 medication. At POM six, 73% of patients required no medications for IOP control, and 41% of those treated achieved IOP ≤ 12 mmHg [Table 4].

##  DISCUSSION

The threshold for surgical treatment of glaucoma has been lowered with the advent of microinvasive glaucoma surgery (MIGS). These procedures offer low-risk, relatively standardized, and highly reproducible techniques for lowering IOP without the financial burden, hazards, and compliance pitfalls of medications. Many of these procedures can be combined with cataract surgery without increasing the risk above standard phacoemulsification.^[12]^ Still, the cost of MIGS devices makes these procedures inaccessible to a large number of patients and surgeons around the world.

The BANG is a cost-effective alternative to many TM-based glaucoma procedures. It utilizes a standard hypodermic needle modified by the surgeon in the fashion of a reverse cystotome to completely excise a segment of TM. A standard cystotome, with its single cutting surface, is not capable of tissue excision without multiple passes. Given the fragility of the TM, multiple passes with any sharp object may cause tissue laceration or fragmentation rather than complete excision. The bevel and lumen of the hypodermic needle combine to form two cutting edges—in effect creating a “double blade” goniotome capable of excising tissue *en bloc* [Figure 2]. The dorsal portion of the bent needle acts as a guard, preventing incision of the posterior wall of Schlemm's canal and helping maintain the plane of excision. The width of the needle's “double blade” is titratable—simply place the bend near the proximal end of the bevel for a wider cutting surface. Similar procedures using a specialized blade have been shown to decrease IOP up to 26.2% over 12 months.^[23]^ While longer follow-up is needed, the IOP-lowering effect of the BANG appears comparable at six months.

In this study, many glaucoma patients were controlled with topical medications prior to surgery—hence, the relatively low preoperative IOP. Each patient with a visually significant cataract and glaucoma controlled with medications was offered a combined phacoemulsification/BANG in an effort to reduce their drop burden. Other patients presented from their referring provider as glaucoma and cataract evaluations, but had not yet been started on topical medication. Upon diagnosis of glaucoma and visually significant cataract, the patient was offered a combined surgery in an effort to achieve IOP control surgically.

Existing TM-based MIGS implants and surgical instruments can be expensive. Many practices, especially in the developing world, do not have the ability to spend hundreds of US dollars on a single-use instrument with mild-to-moderate IOP-lowering effect. Many financially stable practices would welcome the opportunity to lower a glaucoma patient's medication burden via a cost-effective, low-risk, and implant-free MIGS procedure. A recent Internet search showed that a 100-count box of 25-gauge 5/8 inch needles costs approximately 11 US dollars. Additionally, most operating venues already have 25-gauge hypodermic needles at their disposal, making this procedure readily available to the angle surgeon. In an era when MIGS devices can be made unavailable to the population at a moment's notice, having several viable options for safe, effective IOP lowering is paramount.

In conclusion, the BANG procedure represents an accessible MIGS option, with preliminary outcomes similar to those of much more expensive alternatives. While the early results are promising, a prospective study is underway to further characterize the long-term outcomes of this novel technique.

##  Financial Support and Sponsorship 

This work was supported by an unrestricted grant from Robert Feibel, MD.

##  Conflicts of Interest

No author has financial interest in any material or method discussed.
